# Optimizing Sensor Placement for Temperature Mapping during Ablation Procedures

**DOI:** 10.3390/s24020623

**Published:** 2024-01-18

**Authors:** Francesca Santucci, Martina Nobili, Francesca De Tommasi, Daniela Lo Presti, Carlo Massaroni, Emiliano Schena, Gabriele Oliva

**Affiliations:** 1 Unit of Automatic Control, Universitá Campus Bio-Medico di Roma, 00128 Rome, Italy; f.santucci@unicampus.it (F.S.); m.nobili@unicampus.it (M.N.); 2 Unit of Measurements and Biomedical Instrumentation, Universitá Campus Bio-Medico di Roma, 00128 Rome, Italy; f.detommasi@unicampus.it (F.D.T.); d.lopresti@unicampus.it (D.L.P.);; 3 Fondazione Policlinico Universitario Campus Bio-Medico, Via Alvaro del Portillo, 200, 00128 Rome, Italy

**Keywords:** laser ablation, fiber Bragg grating sensors (FBGs), minimal invasive surgery, sensor positioning, temperature monitoring

## Abstract

Accurately mapping the temperature during ablation is crucial for improving clinical outcomes. While various sensor configurations have been suggested in the literature, depending on the sensors’ type, number, and size, a comprehensive understanding of optimizing these parameters for precise temperature reconstruction is still lacking. This study addresses this gap by introducing a tool based on a theoretical model to optimize the placement of fiber Bragg grating sensors (FBG) within the organ undergoing ablation. The theoretical model serves as a general framework, allowing for adaptation to various situations. In practical application, the model provides a foundational structure, with the flexibility to tailor specific optimal solutions by adjusting problem-specific data. We propose a nonlinear and nonconvex (and, thus, only solvable in an approximated manner) optimization formulation to determine the optimal distribution and three-dimensional placement of FBG arrays. The optimization aims to find a trade-off among two objectives: maximizing the variance of the expected temperatures measured by the sensors, which can be obtained from a predictive simulation that considers both the type of applicator used and the specific organ involved, and maximizing the squared sum of the distances between the sensor pairs. The proposed approach provides a trade-off between collecting diverse temperatures and not having all the sensors concentrated in a single area. We address the optimization problem through the utilization of approximation schemes in programming. We then substantiate the efficacy of this approach through simulations. This study tackles optimizing the FBGs’ sensor placement for precise temperature monitoring during tumor ablation. Optimizing the FBG placement enhances temperature mapping, aiding in tumor cell eradication while minimizing damage to surrounding tissues.

## 1. Introduction

The 1990s witnessed the diffusion of *minimally invasive thermal therapies* (MITTs) as techniques for removing cancer. Nowadays, high-intensity focused ultrasound, microwave, radiofrequency, and laser ablation are broadly adopted in clinical settings for treating a variety of tumors (e.g., kidney, lung, liver, bone, and pancreas), especially for patients who are not good surgical candidates [1,2,3,4,5,6,7,8,9,10]. In the context of these techniques, energy is delivered in the form of ultrasounds, electrical current, or laser light and interacts with biological tissue, resulting in a consequent local application of a cytotoxic temperature [11,12,13]. Indeed, the utilization of hyperthermia enables the achievement of the primary goal of MITTs, focusing on eradicating a targeted volume comprising tumoral cells, all the while minimizing interference with the surrounding tissues. Basically, the amount of damaged tissue is related to the temperature experienced by the cells and the exposure time [14,15,16]. Therefore, knowledge of temperature during the ongoing procedure has been recognized as one of the most important aspects to accomplish the mentioned primary objective of MITTs and for enhancing clinical outcomes. In such scenarios, comprehensive observation of the surgical field is not feasible, prompting the use of supplementary tools, such as sensors, to provide real-time information about the ongoing procedure. The limited visibility and constrained space inherent in minimally invasive procedures necessitate the optimization of the use and placement of additional instruments.

Among several invasive and non-invasive techniques, fiber Bragg grating sensors (FBGs) stand out as a powerful sensing solution, due to their small size, good metrological properties, and immunity from electromagnetic interferences [17,18]. In addition, the possibility to perform multi-point temperature measurements [19,20,21,22] facilitates an accurate reconstruction of the temperature map of the target tissue and its surrounding area. In this regard, how to effectively position the FBGs within the target organ to enhance the accuracy of the temperature map remains an open question. This is witnessed by the increasing number of studies that use different numbers and positions of FBGs for temperature monitoring during MITTs [23,24,25].

Our goal is to tackle this challenge through a rigorous scientific approach. We have introduced a theoretical model adaptable to diverse situations and practical scenarios, aiming to optimize FBG sensor utilization for more precise and tailored applications.

The details of the problem, its significance, and the current state of the art, along with our proposed solution, will be outlined in the following paragraph.

### 1.1. Fundamentals and State of the Art

Temperature control during ablation treatments is a task of crucial importance for three main reasons: (i) to be sure that the cytotoxic level has been reached in the entire tumor mass to guarantee the ablation effectiveness, (ii) to avoid carbonization of the cells closest to the source, and (iii) to avoid reaching a too high temperature in an area beyond the tumor mass boundary that would damage healthy cells. For the above reasons, it is crucial to deploy multiple sensors within the cancer tissue and its surrounding boundary to ensure that the cytotoxic level is adequately reached throughout the entire tumor mass. Additionally, a few sensors should be placed beyond the tumor mass to prevent undesired temperature increases. Specifically, the region near the heating source or any area experiencing significant temperature increments is of utmost importance. Therefore, temperature monitoring close to the heating source is necessary to prevent tissue carbonization while also paying attention to peripheral areas around the tumor mass to create an accurate temperature map.

**Figure 1:**
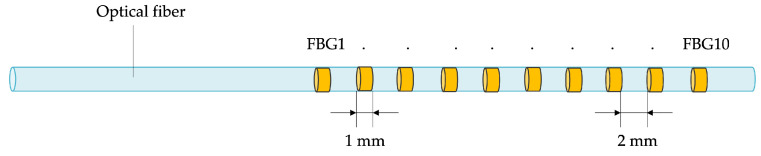
Schematic of an FBG array, illustrating the positioning of individual sensitive elements within a single fiber (not to scale).

Thermometric techniques used for this purpose can be classified as contact-based or contactless methods [24,26]. The contact-based category includes thermocouples, thermistors, fluoroptics, and FBG sensors. Given that current electrical and fluoroptic sensing technologies are confined to single-point temperature measurements, achieving spatial resolution mandates the utilization of multiple probes. For this reason, due to their multiplexing capability, FBGs have emerged as an appealing option for spatial temperature monitoring during MITTs. Indeed, thanks to this feature, it is possible to create arrays that encompass multiple sensitive elements, obtaining multiple temperature points along a line with a single tool (see Figure 1). Then, the use of multiple arrays simultaneously enables measuring a distribution of temperature points not restricted to a straight line, allowing an accurate temperature map reconstruction of the tissue undergoing ablation.

To date, a huge number of studies regarding the use of FBGs for tracking temperature in microwave ablation (MWA), radiofrequency ablation (RFA), laser ablation (LA), and high-intensity focused ultrasound (HIFU) can be found in the literature, testifying to the high potential of this technology in this scenario [25,27,28,29,30,31,32,33,34,35,36,37,38,39,40,41]. The state of the art provides a range of alternatives for employing FBGs during MITTs, including different sizes, number of sensors, distances from the energy source, and areas of tissue covered. Despite this, to date, there are no standard strategies nor established guidelines for positioning FBGs for temperature monitoring during ablation treatments. Some studies place FBGs close to the ablation device to detect the thermal gradient established along the active length and beyond the source tip when FBGs are placed at a higher depth than the ablation applicator [27,32,33,34,40,42,43]. Other studies have suggested that the placement of FBGs encompasses not only the area directly adjacent to the heat source but also the surrounding periphery. In these studies, FBGs are strategically placed at various depths and distances from the energy source, thus obtaining temperature profiles related to a large tissue area [25,28,29,31,33,35,36,37,38,39,41,44]. This approach has the advantage of detecting temperature changes throughout the surface exposed to cytotoxic changes and potential undesirable changes in the surrounding healthy areas. In this case, custom-made sensor holders (e.g., a plexiglass or perspex box) with several holes at fixed distances from the applicator were used to carefully control the FBGs positioning [36,40]. The distances covered by the FBGs in these studies span from a few mm to 30 mm in the longitudinal and transversal directions to the energy source. However, none of these studies performed a thorough investigation to support the chosen distances and to optimize the sensor distribution. Hence, the literature lacks in-depth research focused on optimizing sensor placement for an accurate 3D temperature reconstruction. However, certain factors remain the same for specific procedures. In fact, when using the same applicator and considering the same organ, the heat distribution remains consistent.

To the best of our knowledge, how to optimize the FBGs positioning during MITTs is still an open question, tackled for the first time in this study. Indeed, determining how to position these sensors to achieve the best monitoring results while minimizing the number of tools used remains unexplored from a theoretical standpoint. Historically, this aspect has been approached pragmatically, with arrays inserted based on the physician’s experience. Major implications and potential benefits arising directly from the optimization of the FBG placement during MITTs include higher spatial resolution and improved accuracy in map reconstruction. Both these aspects represent a powerful instrument to gain in-depth information on the ablation area extension and pinpoint the identification of under- and over-treated regions. By doing so, they contribute to achieving more effective control over the treatment results, leading to improved outcomes. Filling this gap will improve patient outcomes and reduce treatment-related complications going forward.

### 1.2. Contribution

**Figure 2:**
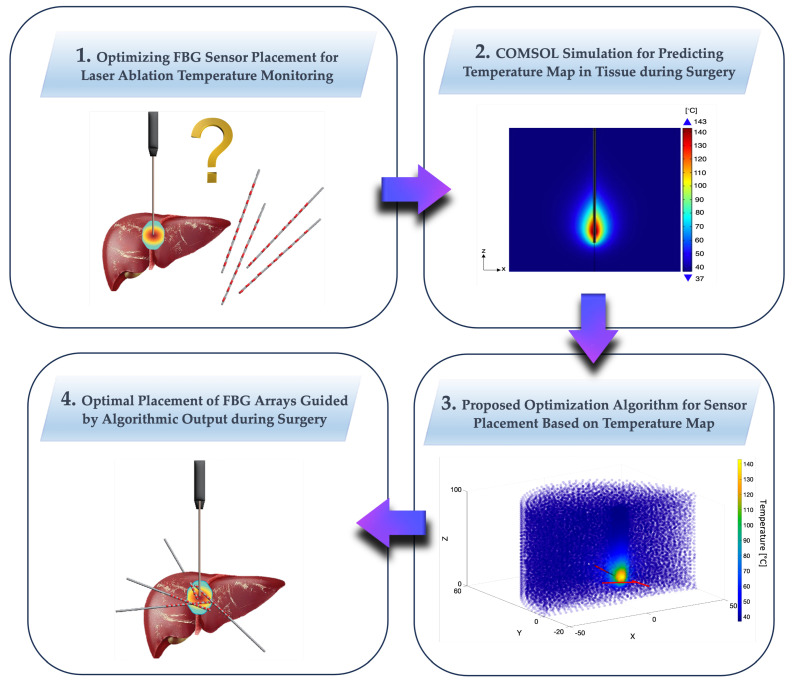
Proposed theoretical optimization method, illustrated through a practical case study. The flow includes (1) addressing the FBGs placement issue for a laser ablation procedure in the pancreas; (2) conducting a COMSOL simulation to predict temperature distribution, resulting in a map of predicted temperatures; (3) utilizing the proposed optimization method to determine optimal sensor positions based on the temperature map for effective temperature monitoring during the procedure; and (4) obtaining the coordinates for the sensor positions.

The aim of this study is to tackle the important problem of optimizing the thermal sensors’ positioning during ablation treatments to obtain an accurate temperature map. In particular, in scenarios involving minimally invasive laser procedures, the comprehensive observation of the surgical field is not feasible. This limitation necessitates the use of supplementary tools, such as sensors, to provide real-time information about the ongoing procedure. The inherent challenges of limited visibility and constrained space in minimally invasive procedures underscore the need for optimization in the use and placement of additional instruments. FBGs are extensively utilized to control temperature distribution during treatment, with the primary objectives of burning all tumor cells, preventing the carbonization of cells near the laser tip due to excessively high temperatures, and avoiding damage to cells outside the tumor area. It is crucial to have the right number of sensors positioned strategically to monitor how and where the temperature is increasing. Historically, in research studies, FBGs have been placed somewhat arbitrarily, often near the laser tip, parallel to the applicator, to capture temperatures at various depths. However, no theoretical framework has been established to determine the optimal sensor placement based on predictive temperature simulations, considering the specific applicator and organ. This gap in the research is partly due to the reliance on experiential practices and the mathematical theorization required. While multiple approaches are feasible, we contend that optimal optimization involves addressing two objectives: maximizing the number of captured temperatures while strategically avoiding sensor overlap in the same area for practical feasibility. Furthermore, identifying optimal points allows for the judicious use of fewer sensors, placed strategically to achieve the same monitoring goals. The theoretical method of optimization proposed has been demonstrated through the application to a practical case in this study, whose approach is summarized graphically in its flow in Figure 2: (1) we consider the problem of the FBGs placement for a laser ablation procedure in the pancreas; (2) a simulation is conducted using COMSOL to predict the temperature distribution during the procedure, thereby obtaining a map of predicted temperatures; (3) the proposed optimization method is utilized to define, based on the temperature map, where sensors should be positioned to optimally monitor temperatures during the actual procedure; and (4) the coordinates for the sensors’ positions are then obtained.

More in detail, we consider the optimal placement of several FBG arrays, each encompassing a number of sensors, aiming to identify the best distribution of the sensors over the FBG-array and the best placement of the FBG-array in the three-dimensional space. Our approach is guided by information about the temperature map expected for the tumor ablation procedure, and our optimization formulation aims to place the FBG-arrays according to a combination of two clashing objectives: on the one hand, we aim at placing the sensors in a way that guarantees to measure a wide variety of different temperatures, i.e., we aim at maximizing the variance of the expected temperatures measured by the different sensors; on the other hand, in order to avoid the sensors being placed too close to each other, we aim at maximizing the squared sum of the distances among each pair of sensors. The two objectives are mediated by a parameter that allows for identifying different trade-offs among the objectives, e.g., prioritizing the collection of diverse temperature values or the geographical dispersion of the sensors. From a technical standpoint, we formulate our optimization problem in terms of a nonlinear optimization formulation. Notably, the proposed problem is nonconvex, and its solution represents a nontrivial challenge, thus calling for approximation schemes. This paper is complemented by a simulation campaign that numerically demonstrates the effectiveness of the proposed approach.

## 2. Materials and Methods

In this section, we develop the proposed optimization formulation that will be the cornerstone for the decision-support tool developed in this paper. Notably, the problem is nonconvex, and its solution represents a nontrivial challenge, thus calling for approximation schemes.

### 2.1. Key Optimization Concepts

This subsection aims to cover some fundamental concepts in optimization that will be used later in this paper; the interested reader is referred to books such as [45] for a comprehensive overview. Consider a function f(x), $f:R^{n}\to R$ where $x\in R^{n}$. The function f(x) is *convex* if

(1) f(αx+(1−α)y)≤αf(x)+(1−α)f(y)

for all α∈[0, 1] and all $x,y\in R^{n}$ and is said to be *nonconvex* otherwise. If the above inequality is satisfied as a strict inequality, the f(·) is said to be *strictly convex*. An optimization formulation is, typically, in the form

(2) $$\begin{array}{l} \min_{x\in R^{n}}\;\;\;\;f\left(x\right),\\ \mathrm{subject}\;\mathrm{to}\;\\ \left\{\begin{array}{cc} h\left(x\right)=0_{m}, & \\ g\left(x\right)\geq 0_{l}. & \end{array}\right. \end{array}$$

where $g:R^{n}\to R^{m}$ and $h:R^{n}\to R^{l}$. In other words, the above optimization formulation aims at finding a solution $x^{*}\in R^{n}$ that is *feasible*, i.e., it satisfies the equality and inequality constraints and is *optimal*, i.e., all other feasible solutions in a neighborhood of $x^{*}$ have a higher value of f(·). If the neighborhood coincides with all $R^{n}$, then $x^{*}$ is said to be *globally optimal*; otherwise, the solution is said to be *locally optimal*. Notably, if f(·), g(·), h(·) are convex (a vector-valued function is convex if each of its components is convex), then the problem itself is said to be convex (while it is nonconvex otherwise) and has a globally optimal solution. In particular, assuming the problem to be convex, such a solution is globally optimal if and only if the so-called *Karush–Kuhn–Tucker* (KKT) conditions hold true. If, moreover, f(·) is strictly convex, then the global optimal solution is unique. If the problem is convex, then finding a solution can be regarded as a manageable task, as several exact solvers can be relied upon. If, conversely, the problem is nonconvex, then the KKT conditions are only necessary for optimality, and the task of finding a good solution is greatly complicated. In this latter case, a popular strategy is to resort to approximated solvers (e.g., generating random solutions and selecting the best one among those that are feasible), with the aim to empirically identify a good solution in a reasonable time. However, the quality of the solution found is dependent on the structure of the particular problem, as well as on the dimension of the problem in terms of the dimension *n* of the sought variables.

### 2.2. Problem Formulation

Let us consider a situation where there is a region of interest $X\subset R^{3}$ and a function $f:R^{3}\times R\to R$ that provides the expected temperature for a point p∈X at a given time t≥0. In particular, in the following, we consider a set of discrete-time instants $t_{1},\ldots ,t_{T}$.

**Figure 3:**
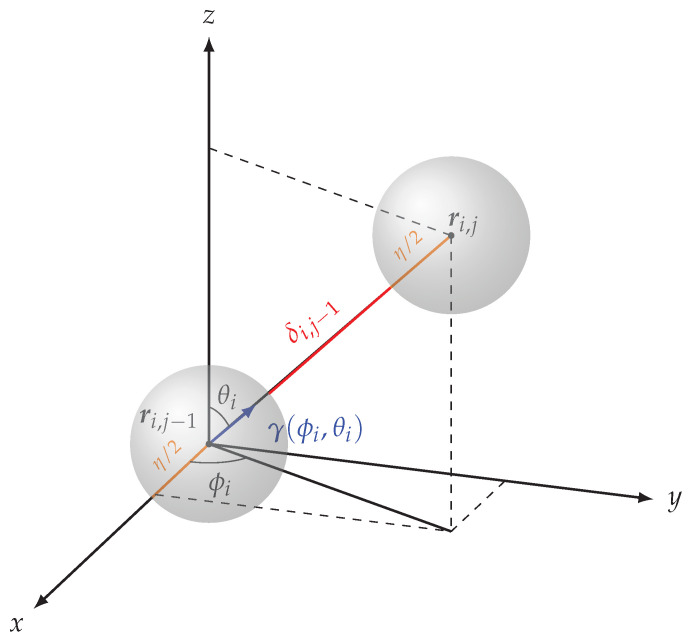
Relation between the *j*-th and (j−1)-th sensors in the same FBG-array. The position $r_{ij}$ is obtained from $r_{i,j-1}$ by moving $\epsilon +\delta_{i,j-1}$ (i.e., the distance $\delta_{i,j-1}$ between the two spheres, plus the radius ϵ/2 of each sphere) toward $r_{i,j}$, i.e., along the direction described by the unit-length vector $\gamma (\phi_{i},\theta_{i})$. Iterating, the position of each sensor in an FBG-array is uniquely determined by the diameter η, by the position of the first sensor, by the angles $\theta_{i},\;\phi_{i}$, and by the distances $\delta_{i,j}$.

Moreover, let us consider a set of *m* FBGs-arrays of maximum length λ and let us assume that each FBGs-array features *n* sensors. Let us use $r_{i,j}\in R^{3}$ to denote the position of the center of mass of the *j*-th sensor belonging to the *i*-th FBGs-array. In particular, for the sake of simplicity, we model each sensor as a sphere with a diameter equal to η. Finally, let $\delta_{i,j}\geq 0$ denote the distance between two consecutive sensors *j* and j+1 belonging to the *i*-th FBGs-array. In more detail, being the sensors modeled as spheres, $\delta_{i,j}$ represents the distance between their boundaries. Interestingly, the actual distance between the centers of mass must also include the radius (i.e., η/2) of both spheres and is thus equal to $\eta +\delta_{i,j}$. In this view, it is convenient to show that the positions $r_{i,1},\ldots ,r_{i,n}$ of the sensors in the same FBGs-array are uniquely determined by *n*, η, by the terms $\delta_{i,1},\ldots ,\delta_{i,n-1}$, and by five additional parameters: the position

(3) $$r_{i,1}={\left[r_{i,1}^{x},r_{i,1}^{y},r_{i,1}^{z}\right]}^{T}$$

of the center of mass of the first sensor in the FBGs-array and the angles $\theta_{i},\phi_{i}$ describing the relative bearing in the spherical coordinates of all the other sensors with respect to the first one. In fact, by using some algebra, we observe that the position $r_{i,j}$ of the center of mass of the *j*-th sensor in the array can be obtained from the (j−1)-th one by moving $\eta +\delta_{i,j}$ along the segment in $R^{3}$ that joins $r_{i,j}$ and $r_{i,j-1}$; in other words, we can express the position of the sensors j∈{2,…,n} belonging to the *i*-th FBGs-array according to the following recursive definition

(4) $$r_{i,j}=r_{i,j-1}+\left(\eta +\delta_{i,j-1}\right)\underset{\gamma (\phi_{i},\theta_{i})}{\underset{︸}{\left[\begin{array}{c} \cos\left(\phi_{i}\right)\sin\left(\theta_{i}\right)\\ \sin\left(\phi_{i}\right)\cos\left(\theta_{i}\right)\\ \cos(\theta_{i}) \end{array}\right]}},\;\;j\in \left\{2,\ldots ,n\right\},$$

where $\gamma (\phi_{i},\theta_{i})$ is the unit-length vector oriented in the direction from $r_{i,j-1}$ to $r_{i,j}$, as encoded by the angles $\phi_{i},\theta_{i}$ (see Figure 3).

Interestingly, defining the set $P_{i}$ as

(5) $$P_{i}=\left\{r_{i,1}^{x},r_{i,1}^{y},r_{i,1}^{z},\theta_{i},\phi_{i},\delta_{i,1},\ldots ,\delta_{i,n-1}\right\},$$

we can iterate Equation (4) and express the position of each sensor in the *i*-th FBGs-array as a function of *n*, η, and the parameters in $P_{i}$, i.e.,

(6) $$\begin{array}{cc} r_{i,j} & =r_{i,j-1}+\left(\eta +\delta_{i,j-1}\right)\gamma \left(\phi_{i},\theta_{i}\right)\\ \; & =r_{i,j-2}+\sum_{h=j-2}^{j-1}\left(\eta +\delta_{i,j-1}\right)\gamma \left(\phi_{i},\theta_{i}\right)\\ \; & =\ldots \\ \; & =r_{i,1}+\sum_{h=1}^{j-1}\left(\eta +\delta_{ih}\right)\gamma \left(\phi_{i},\theta_{i}\right),\;j\in \left\{2,\ldots ,n\right\}. \end{array}$$

Notice that, for the positions to be compatible with the length λ of the FBG-array, we need to enforce that all terms $\delta_{i,j}\geq 0$ and that

(7) $$∥r_{i,1}-r_{i,n}∥+\eta \leq \lambda ;$$

in fact, the length of the FBG-array is the distance between the centers of mass of the first and last sensor, plus η/2 to account for the radius of the first one and an additional η/2 to account for the radius of the last one.

By plugging Equation (6) into Equation (7), we have that the above constraint is equivalent to requiring that

(8) $$∥\sum_{h=1}^{j-1}\left(\eta +\delta_{ih}\right)\gamma (\phi_{i},\theta_{i})∥\leq \lambda -\eta .$$

Lastly, let Y⊆X denote an *interdicted zone*, i.e., a region where the FBG-arrays should not be placed (e.g., due to the vicinity to the emitter); in this view, we want the position of each sensor to satisfy

(9) $$r_{i,j}+S_{\eta }\subseteq X,$$

where $S_{\eta }$ is a sphere of radius η centered in the origin. At the same time, we want the entire FBG-arrays not to belong to Y; in order to model this requirement, we consider

(10) $$H_{i}\left({\left\{r_{ij}\right\}}_{j=1}^{n}\right)=\mathrm{convex}-\mathrm{hull}\left\{r_{ij}+S_{\eta }\;|j\in \left\{1,\ldots ,n\right\}\right\},$$

i.e., the convex hull of the *n* spheres representing the sensors that belong to the same FBG-array, and, for simplicity, we enforce that

(11) $$B_{i}\left({\left\{r_{ij}\right\}}_{j=1}^{n}\right)⋂Y=Ø,$$

where $B_{i}\left(\cdot \right)$ is the bounding box of $H_{i}\left(\cdot \right)$ in $R^{3}$, i.e.,

(12) $$B_{i}\left({\left\{r_{ij}\right\}}_{j=1}^{n}\right)=\mathrm{bounding}-\mathrm{box}\left(H_{i}\left({\left\{r_{ij}\right\}}_{j=1}^{n}\right)\right).$$

Our aim is to maximize two possibly conflicting objectives. As for the first objective, we aim to maximize a weighted sum over the different time instants and over the different FBG-arrays of the variances $\xi_{i}\left(t\right)$ of the temperature values that correspond to the selected sensors’ positions $r_{i,j}$ for each FBG-array, i.e., the aim is to maximize

(13) $$\begin{array}{c} g\left(\left\{r_{ij}\right\}\right)=\sum_{z=1}^{T}\alpha_{z}\sum_{i=1}^{m}\underset{\xi_{i}\left(t_{z}\right)}{\underset{︸}{\frac{1}{n}\sum_{j=1}^{n}(f\left(r_{ij},t_{z}\right)-\frac{1}{n}\sum_{k=1}^{n}f\left(r_{ik},t_{z}\right){)}^{2}}}, \end{array}$$

where we use $\left\{r_{ij}\right\}$ to denote the terms $r_{ij}$ for all *i* and *j*, while each coefficient $\alpha_{z}\geq 0$ characterizes the importance or weight of the variance of the temperatures at the *z*-th time instant. Regarding the second objective, we aim to place the sensors in positions that are as far apart from each other as possible, in order to improve the coverage of the region X of interest. In this view, the second objective amounts to the sum of the square Euclidean distances among all pairs of sensors that do not belong to the same FBG-array, i.e.,

(14) $$h\left(\left\{r_{i,j}\right\}\right)=\sum_{i=1}^{m}\sum_{j=1}^{n}\sum_{u=1,\;u\neq i}^{m}\sum_{v=1}^{n}{∥r_{i,j}-r_{uv}∥}^{2}.$$

In particular, for the sake of combining the two objectives, we suitably normalize (we normalize the first objective, dividing it by its maximum possible value (i.e., assuming that, at each time instant, the sensors in each FBG-array measure the available temperature values that result in the largest possible variance; as for the second objective, again, we normalize it with respect to its maximum possible value (i.e., assuming that the distance of each pair of sensors coincides with the maximum possible distance in the space of interest)), so that the resulting normalized values $\bar{g}\left(\left\{r_{ij}\right\}\right)$ and $\bar{h}\left(\left\{r_{i,j}\right\}\right)$ both belong to [0, 1]. Notably, our strategy to mediate between the two objectives is to consider a convex combination based on a parameter β∈[0, 1], i.e., we aim to maximize

(15) $$\beta \bar{g}\left(\left\{r_{ij}\right\}\right)+\left(1-\beta \right)\bar{h}\left(\left\{r_{ij}\right\}\right),$$

which can be equivalently obtained by minimizing (it can be easily shown that maximizing f(x) is equivalent to minimizing −f(x)).

(16) $$-\left(\beta \bar{g}\left(\left\{r_{ij}\right\}\right)+\left(1-\beta \right)\bar{h}\left(\left\{r_{ij}\right\}\right)\right).$$

In conclusion, our optimization formulation can be summarized as follows.

**Problem 1.**  Let $f:R^{3}\times R\to R$ be given and consider a finite and discrete set $t_{1},\ldots ,t_{T}$ of time instants. Let coefficients $\alpha_{z}\geq 0$ be given for z=1,…,T. Moreover, let there be *m* FBG-arrays with *n* sensors each, and let η denote the diameter of each sensor. Find the parameters $r_{i1}\in R^{3}$, $\theta_{i},\;\phi_{i},\;\delta_{i,1},\ldots ,\delta_{i,n-1}\in R$ for each FBGs-array that solves

(17) $$\begin{array}{l} \min-\left(\beta \bar{g}\left(\left\{r_{ij}\right\}\right)+\left(1-\beta \right)\bar{h}\left(\left\{r_{ij}\right\}\right)\right),\\ \mathrm{subject}\;\mathrm{to}\\ \left\{\begin{array}{cc} r_{i,j}=r_{i1}+\sum_{h=1}^{j-1}\left(\eta +\delta_{ih}\right)\gamma \left(\phi_{i},\theta_{i}\right), & \;\;\;\;\;\;\forall i,\forall j>1,\\ ∥\sum_{h=1}^{j-1}\left(\eta +\delta_{ih}\right)\gamma (\phi_{i},\theta_{i})∥\leq \lambda -\eta , & \forall i,\\ r_{i,j}+S_{\eta }\subseteq X, & \forall i,j\\ B_{i}\left({\left\{r_{ij}\right\}}_{j=1}^{n}\right)⋂Y=\emptyset , & \forall i\\ \delta_{i,j}\geq 0, & \forall i,\;\forall j\leq n-1. \end{array}\right. \end{array}$$

We reiterate that, within the above problem, we aim to place each sensor in a position $r_{i,j}$ in order to maximize a convex combination of two possibly conflicting (the objectives are possibly conflicting, because the variance of the temperature could be maximized with positions that fail to optimize the second objective or vice versa; for this reason, the two objectives are reconciled by considering a trade-off, driven by the parameter β) objectives: (i) a weighted sum over all time instants of the variance of the corresponding temperature and (ii) the sum of all squared Euclidean distances among the sensors belonging to different FBG-arrays. Moreover, we want sensors on the same FBG-array to lie on the same segment of length at most λ, and we want the center of mass of consecutive sensors *j* and j+1 to be spaced by $\eta +\delta_{i,j}$ along such segment, and we want the sensors to belong to X while the entire FBG-arrays must not belong to Y.

Notably, the above optimization formulation is nonconvex: in fact, the constraints feature trigonometric functions of the angles $\theta_{i},\;\phi_{i}$, which are not convex in nature. Moreover, within the proposed formulation, we are minimizing a negative quadratic function, which is concave and thus nonconvex.

### 2.3. Approximated Solution Strategy

We point out that the main challenges for attempting to solve the problem are (i) the fact the problem formulation is nonconvex and (ii) the fact that the temperature is only available at discrete locations. Regarding point (ii), we have that, as discussed later in this paper, the simulation was performed in COMSOL™, and then, from the temperature map simulated for various time instants, a discrete set of points was sampled.

Given the complexity of the problem, in this paper, our strategy to calculate an approximated solution is to resort to an approximated solver. In particular, suppose that the temperature is only available at discrete locations in X; our strategy to evaluate the temperature measured by a sensor at a given position is to select the known temperature of the point that is closest to the selected sensor location.

In other words, the value assumed by the objective function has to be computed in an algorithmic way, without a closed form. Also, the nonconvex nature of the problem makes classical approaches such as gradient descent useless.

Further to that, we observe that, given the symmetry of the setting considered in this paper, the simulated tridimensional temperature profile could be effectively approximated by considering the bidimensional section along the x-z plane and then by rotating it around. In this view, in order to save computational resources, we opted to consider the bidimensional data and, while comparing distances, we considered the projection of the tridimensional points on the x-y plane.

In order to address these challenges, we use the MIDACO optimization software (version 5.0), which implements an extension of the evolutionary Ant Colony Optimization meta-heuristic [46,47,48,49,50] and which was developed especially for highly nonlinear real-world applications. See [51] or [52] for a focus of the performance of the MIDACO software with respect to the state of the art.

Note that the suggested strategy is independent of a particular solver, but the nonconvex nature of the optimization problem, and the algorithmic description of the objective function, suggest an evolutionary approach, like genetic algorithms [53,54].

In particular, the optimization with MIDACO was conducted on an Intel®Core(TM) CPU i9-9900K @ 3.60GHz with a RAM of 64 GB. The CPU runtime for the optimization was fixed to around 3 h. All the MIDACO parameters were used at their default values, which means that a feasibility accuracy of 0.001 was used for all individual constraints listed in Equation (17).

## 3. Results

After outlining the proposed theoretical method for optimizing the placement of FBG sensors for temperature monitoring, this section focuses on applying the method to a case study, detailing the application steps, and presenting the results. Specifically, in Section 3.1, ‘*Generation of Reference Temperature Maps using Simulation Software*’, we explain the simulation process to generate a predictive map of temperatures, taking into account the type of applicator used and the characteristics of the organ undergoing ablation; in Section 3.2, ‘*Investigation of Algorithm Performance: Application to Simulated Temperature Map*’, we showcase the application of the theoretical method to this specific case and illustrate the results in terms of FBG sensor positions. Additionally, we provide details on key parameters for utilizing the method, highlighting its versatility across various applications.

### 3.1. Generation of Reference Temperature Maps Using Simulation Software

To replicate temperature distributions experienced in real-world scenarios, we conducted simulations of MWA (microwave ablation) treatments in liver tissue using the COMSOL™ Multiphysics environment based on the finite element method. These simulations considered different settings. Specifically, three different power levels (i.e., 20 W, 30 W, and 40 W) were applied for the same treatment duration of 5 min. As commonly encountered in the state of the art, a cylindrical domain (i.e., radius: 50 mm and height: 100 mm) was used to simplify the actual organ shape, assuming it possesses homogeneous and isotropic properties [29,55,56,57]. In our simulations, as a boundary condition, we set a temperature of 37 °C at the extremities of the domain. This condition was imposed to simulate the scenario where, far from the heat source, the tissue does not experience a significant temperature increase, effectively replicating the natural state where distant regions remain at normal body temperature. Given the cylindrical symmetry of the temperature profile of the region of interest, we opted to reduce the dimensionality of the input data by sampling temperatures only along the x-z plane (i.e., the plane obtained when the y coordinate is equal to zero) and then projecting points in three dimensions (i.e., the sensors’ coordinates) on the plane in order to associate them to a temperature measure. Table 1 lists the physical and magnetic properties assigned to the simulated volume, either based on data from the existing literature or available in the software library [58,59].

The MW applicator responsible for energy delivery consisted of a coaxial antenna with three concentric layers, an inner conductor, a dielectric layer, as well as an outer conductor for a total diameter of 1.79 mm, positioned in the middle of the domain [60]. The antenna’s metal components were excluded from the computational domain and modeled under perfect electric conductor conditions to minimize the computation expenses. Further, 2.45 GHz was selected as the MW operating frequency because it is widely used for MWA systems [61,62]. The temperature distribution resulting from the MW energy–tissue interaction was determined by solving the bioheat equation, stated as follows [63]:

(18) $$\rho \cdot C\cdot \frac{\partial T}{\partial t}=\nabla \left(k\cdot \nabla T\right)+Q_{b}+Q_{s}+Q_{met}$$

where C represents the specific heat, k denotes the thermal conductivity, and T the temperature of the tissue. $Q_{b}$, $Q_{s}$, and $Q_{met}$ refer to three distinct heat contributions per unit volume (measured in W·m^−3^). Each term is detailed below:

- $Q_{b}$ represents the heat flow resulting from the blood perfusion in the tissue. It is determined by parameters, such as the blood density ($\rho_{B}$), the specific heat of the blood ($c_{b}$), and the rate of the blood perfusion ($\omega_{b}$) listed in Table 2.
- $Q_{s}$ represents the heat generated by the MW source, known as dielectric heating, and caused by the continuous reorientation of water molecules in response to the applied magnetic field.The value of Qs can be expressed as a function of the tissue density and the specific absorption rate (SAR), which quantifies the amount of electromagnetic energy absorbed per unit mass of tissue.
- $Q_{met}$ accounts for the heat produced by the metabolic processes within the tissue. This contribution is considered negligible compared to the other heat contributions in the equation.

The dense mesh numerical simulations were carried out on a workstation with an Intel Xeon Silver 4210 2.20 GHz processor and 128 GB of RAM, enabling to gather 3D temperature distributions at different time instants for each simulated condition of probe power (i.e., 20 W, 30 W, and 40 W). The cylindrical domain used for the three-dimensional simulations had dimensions of 100 mm in diameter and 100 mm in height. It was discretized into 3,071,259 elements, and the simulations took approximately 18 min to compute. A finer mesh was specifically applied near the MW applicator, as this tissue region experiences higher temperatures and thus requires enhanced resolution for accurate modeling.

In particular, for the scenario, we considered 21 different temporal instants from t=0 s to t=300 s, with a 15 s sampling time. For each considered time instant, we sampled 17,169 points on the x-z plane, using a grid-like spatial sampling strategy.

### 3.2. Investigation of Algorithm Performance: Application to Simulated Temperature Map

The model we have developed for this problem in the MATLAB™ environment takes as input a series of parameters that can be adjusted and tailored to each individual ablation case. This ensures a high adaptability of the model to diverse situations and the specific requirements of the intervention. In fact, specific constraints dictated by the application scenario could also be present, such as the number of available FBG arrays for the intervention setup, which, in turn, may depend on the capabilities of the optical interrogator, or the number of FBGs on each individual probe, which is also contingent upon the fabrication process. Consequently, we have opted to parameterize a series of variables that can be defined as input by the user prior to the intervention, based on the characteristics of the specific case. These adjustable parameters include the number of FBG arrays, the number of FBG sensors per array, the total length of the FBG array, the dimensions of each individual FBG sensor, the minimum distance from the emitter for sensor placement, the number of temporal instances considered, the minimum distance between arrays, the designated volume for array placement, and the trade-off value between the two objectives (which determines the weighting of one over the other, should a preference for one objective be desired in the solution definition), as well as the coefficients for the different time instants. Additionally, other parameters can be set to regulate the operation of the MIDACO software, such as the maximum number of iterations.

Granting users the ability to set these parameters ensures the significant versatility of the algorithm, allowing its application in highly varied scenarios, ranging from experimental setups, tumor types, and tissue heat diffusivity to manufacturing constraints for instruments, among others.

In the context of this study, all the simulations were conducted considering the following values for the aforementioned settable parameters: a maximum length of 20 mm per array; four arrays of FBG sensors; 10 FBG sensors for each array; a minimum distance of 0.1 mm from the emitter; and no fixed distance between the sensors. A greater emphasis is given to the second objective with respect to the first one (i.e., β=0.99), discriminating solutions based on the distance between the different FBG arrays; two temporal instances were extracted from the different instances obtained during the simulation of the treatment. For what concerns the MIDACO settings, the maximum number of iterations was set to 100,000.

**Figure 4:**
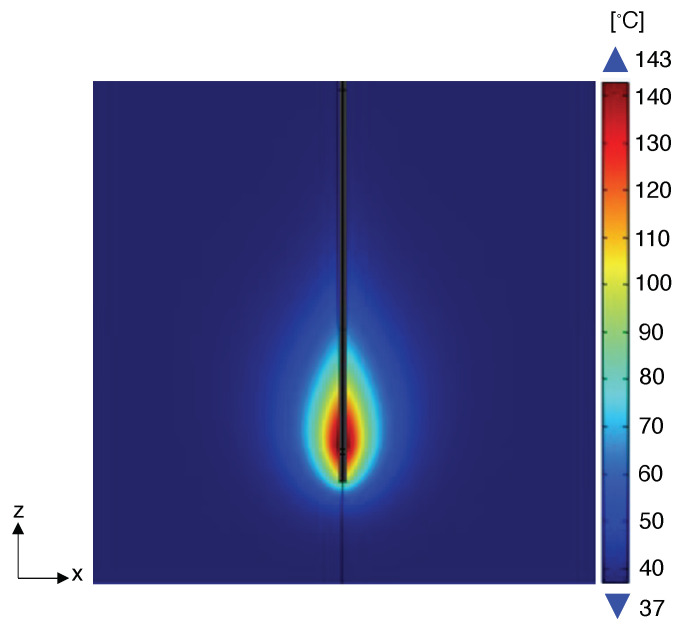
Temperature distribution obtained for numerical simulation carried out at 20 W in correspondence of the final ablation time (i.e., 5 min), and visualized in the plane containing the MW antenna as an example.

Furthermore, in the simulations, a minimum distance of 0.1 mm from the applicator was considered for the placement of the arrays, as there is a physical constraint that the sensors cannot overlap with the emitter. In other words, we assume the interdicted zone Y considered in our formulation is a cylinder of radius 0.1 mm with a central axis that corresponds to the applicator (i.e., the central axis of the cylinder is the z-axis in Figure 4).

Furthermore, to account for the physical constraint that each individual FBG sensor has its own physical size, the sensors are treated as spheres with a diameter of 1 mm in order to incorporate their spatial volume in the arrangement within the FBG array.

From the COMSOL simulation, the temperature data points and their corresponding 3D spatial coordinates were extracted at various temporal instances, precisely at two time points, a middle point at 150 s and the last instant at 300 s, coinciding with the end of the ablation procedure and thus with the actually burnt area. Based on the set of points representing the temperature distribution in space, in order to reduce the computational burden, we chose to consider an xz plane that signifies a cross-section within the initial cylinder. From this selected plane, we downsampled the available data, obtaining 17,169 samples. Leveraging the cylindrical symmetry of the data points, it is possible to posit that the 3D points constitute a projection of the points represented in the examined xz plane. Specifically, while evaluating the temperature associated with a point in the three-dimensional space, we chose to project the point onto the considered plane and to select the temperature point that was closest to the projected point. The considered section is the longitudinal cylinder section parallel to the applicator, and its short side represents the maximum diameter of the cylinder’s base. In this way, we ensure a good covering of the different temperatures.

**Figure 5:**
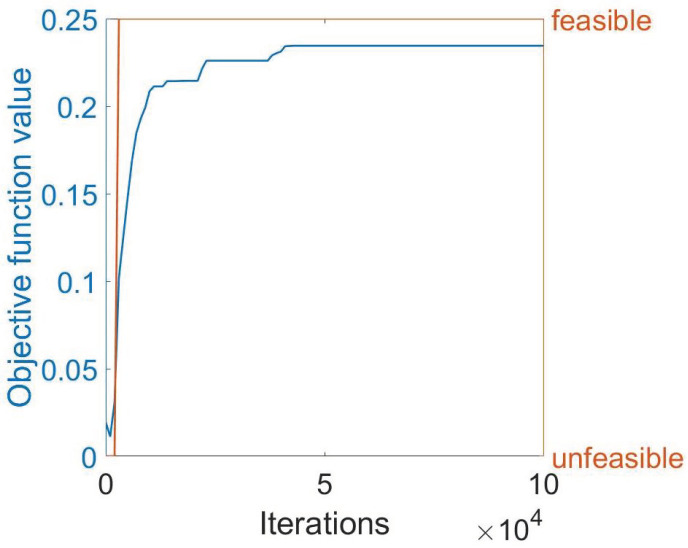
Illustrating the behavior of the objective function as it stabilizes to a specific value after a certain number of iterations. The blue curve represents the objective function, while the orange curve shows whether the solution found at a given iteration is feasible or not.

Because the problem, as previously discussed, amounts to a nonconvex optimization formulation, the exact solution was not possible and an approximate solution was achieved numerically using the MIDACO software. In particular, Figure 5 shows the numerical results obtained by MIDACO during the computation of the identified solution. For instance, we present the case of the objective function obtained for the simulation with β=0.99, four FBG arrays, and power equal to 20 W. Specifically, we observe that, after around 50,000 iterations, MIDACO is able to find a feasible solution (i.e., a solution that satisfies all the required constraints). As the time grows, MIDACO improves such a solution reaching a plateau value of 0.23451010 after around 44,000 iterations. The value thus found remains stable for 56,000 iterations, i.e., until the end of the approximated computation procedure. This supports the conclusion that MIDACO was able to identify a local maximum.

**Figure 6:**
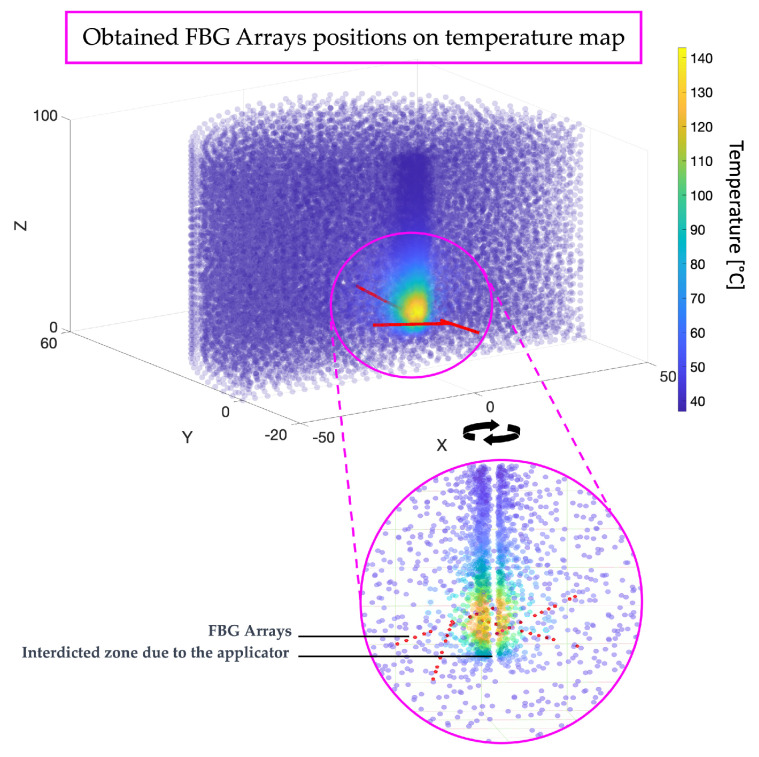
Illustrating the positions of FBG arrays on the temperature map resulting from the simulation. Red points indicate the positions of individual FBGs within the arrays, arranged along lines defining each FBG array.

At the conclusion of the simulation, in addition to the value of the objective function at regular intervals, the positions of all the FBG sensors in each array are provided as the results in terms of the spatial coordinates x, y, and z. This allows for the visualization of the solution with respect to the temperature distribution volume from the COMSOL simulation, as depicted in Figure 6, enabling an assessment of the sensor placements relative to varying temperatures. Specifically, the angular coordinates of the first FBG in each array are determined, followed by the distribution of the others along a line at varying distances (see Table 3. Cartesian coordinates of all the FBGs in all the arrays for the specific case of this simulation are presented in Table 4.

## 4. Conclusions and Future Work

Our approach to developing the FBG sensor placement model was proposed to address a gap in the existing literature. Indeed, we observed a lack of rigorously theorized models for optimizing FBG placement in this context. In the absence of established methodologies in the literature, our focus has been on formulating a mathematical model that addresses the fundamental challenges and objectives associated with FBG sensor placement. Our effort has been directed toward the mathematical moderation of the problem and its solution, aiming to optimize the two main objectives sought from sensor usage in this scenario. Our contribution lies in the mathematical rigor applied to formulate a solution to a problem that, to our knowledge, has not been extensively addressed in prior research. The proposed approach has been tested by considering a practical case study.

As the results demonstrate, in this specific application, all the FBG arrays have been placed around the central temperature blob. Indeed, this is the volume zone where there is a greater temperature variance. In contrast, temperatures outside this area tend to vary minimally, consistently hovering around 37 degrees Celsius, as it represents the part of the organ unaffected by heat variation due to the applicator. Conversely, within the tumor mass, it is crucial to have multiple FBGs to capture the significant temperature variations in a confined space. The FBG arrays are not overlapping, and contrary to common practices in the literature, based on human experience, they are placed obliquely, with angles that deviate from the central axis of the applicator. Typically, in the literature, sensors are placed vertically because it is more convenient for the operator, whereas predicting the correct angle of inclination to capture multiple temperatures is more challenging. This outcome can only be achieved through sophisticated software and algorithms like the one proposed here, providing surgeons with precise angles for optimal sensor array placement to effectively monitor ablation temperatures.

In conclusion, the results obtained align with what one might expect from observing the predicted temperature map from the simulation: the provided positions aim to capture as many temperatures as possible, but they do so more effectively than an operator could achieve visually or randomly without precise guidance. Thus, our unique contribution rests in the meticulous application of mathematical rigor to devise a solution for a problem that, to the best of our knowledge, has not received comprehensive attention in previous research.

Future work will be mainly devoted to experimentally testing the proposed approach in a clinical setting. Also, we will investigate the possibility of applying the technique to different clinical procedures. For instance, we will investigate the possibility of orienting the sensor placement based on an assessment of the tumor margins [64] or account for tissue regeneration [65].

### Limitations

One limitation of this study is that due to the positions being at specific precise angles rather than parallel or vertical to the applicator, a certain degree of accuracy in placement will be required. This also implies the need to find a meticulous place for the array placement. However, this limitation, despite lying outside the scope of this study, could potentially be overcome, considering that modern medicine now possesses precision instruments, particularly for surgical and minimally invasive procedures. Surgical robots and similar tools, as well as other simplified devices, can undoubtedly aid in the placement process. Nonetheless, the objective of this study was to provide optimal positions for effectively mapping temperatures, making the best use of a certain number of fiber-optic sensor arrays.

Furthermore, in our study, the traditional Pennes equation for bioheat was used to calculate the temperature distribution by means of a numerical simulation. This model, widely recognized for its simplicity and effectiveness in various applications, is based on the assumption of homogeneous and isotropic blood perfusion. This assumption simplifies the complex nature of blood flow and heat transfer in biological tissues, which are often heterogeneous. Advances in the field have led to the development of modified forms of the Pennes equation for bioheat, incorporating heterogeneous or anisotropic blood perfusion [66]. This new model provides a better understanding of the bioheat transfer, potentially leading to more accurate predictions in scenarios where uniform blood perfusion is not a valid assumption. Future research could benefit from integrating this modified bioheat equation to address this limitation. This approach would allow for subsequent studies to offer a more detailed and accurate representation of bioheat transfer in living tissue, enhancing the applicability of the present research in MITTs.

While our decision to carry out a 2D analysis along the x-z plane was justified by the cylindrical symmetry of the temperature profile and computational efficiency, it is worth noting that this approach may not capture asymmetric heating effects or variations in tissue properties, aspects that could be more comprehensively addressed through a full 3D analysis. In future studies, we aim to explore a full 3D analysis to capture asymmetric heating effects and variations more accurately in tissue properties. This advancement will expand our understanding of thermal patterns in more complex and heterogeneous tissue environments.

Finally, our simulation-based approach for SAR quantification did not incorporate the direct pixel-based analysis from images [67]. This method could enhance the precision of SAR estimations in heterogeneous tissue environments, representing an area for future methodological enhancement.

## Figures and Tables

**Table 1:** Physical and magnetic properties of the simulated liver tissue.

Property	Value
Specific heat	3450 J/(kg·K)
Density	1079 kg/m^3^
Thermal conductivity	0.52 W/m·K
Relative permittivity	43.03
Electric conductivity	1.69 S/m

**Table 2:** Parameter used for the bioheat equation.

Parameter	Value
Blood-specific heat	3639 J/(kg·K)
Blood temperature	37 °C
Rate of blood perfusion	0.0064 1/s
Blood density	1000 kg/m^3^

**Table 3:** Coordinates of the first FBG sensors for each FBG-array.

Array	x	y	z	θ	ϕ
1	1.2284	−10	27.0191	5.4247	3.4017
2	−9.0776	10	36.9959	2.6018	3.9627
3	7.1518	6.9311	14.8389	5.2708	1.7966
4	9.7561	−10	20.1085	6.2832	2.4477

**Table 4:** Coordinates of sensor positions as resulting from the simulation.

Sensor Coordinates			
**Array 1**	**x**	**y**	**z**
**FBG 1**	1.2284	−10	27.0191
**FBG 2**	−0.0143	−8.561	26.5132
**FBG 3**	−0.6492	−7.8261	26.2547
**FBG 4**	−1.4710	−6.8745	25.9202
**FBG 5**	−4.1817	−3.7361	24.8167
**FBG 6**	−5.0684	−2.7094	24.4557
**FBG 7**	−5.9574	−1.6802	24.0938
**FBG 8**	−6.7858	−0.7210	23.7565
**FBG 9**	−7.4927	0.0975	23.4688
**FBG 10**	−10.0000	3.0005	22.4480
**Array 2**	**x**	**y**	**z**
**FBG 1**	−9.0776	10	36.9959
**FBG 2**	−8.4931	9.6498	36.2640
**FBG 3**	−6.8704	8.6775	34.2323
**FBG 4**	−6.1875	8.2684	33.3773
**FBG 5**	−5.3956	7.7939	32.3858
**FBG 6**	−4.6420	7.3423	31.4422
**FBG 7**	−3.0937	6.4146	29.5036
**FBG 8**	−1.8154	5.6487	27.9031
**FBG 9**	−1.0663	5.1999	26.9652
**FBG 10**	−0.4818	4.8496	26.2333
**Array 3**	**x**	**y**	**z**
**FBG 1**	7.1518	6.9311	14.8389
**FBG 2**	6.9088	7.3200	16.8348
**FBG 3**	6.7469	7.5792	18.1649
**FBG 4**	6.4497	8.0548	20.6054
**FBG 5**	6.2684	8.3450	22.0951
**FBG 6**	5.8874	8.9547	25.2242
**FBG 7**	5.7085	9.2411	26.6939
**FBG 8**	5.5040	9.5684	28.3735
**FBG 9**	5.3563	9.8048	29.5871
**FBG 10**	5.2343	10.0000	30.5886
**Array 4**	**x**	**y**	**z**
**FBG 1**	9.7561	−10	20.1085
**FBG 2**	8.6967	−10	20.9899
**FBG 3**	7.6801	−10	21.8357
**FBG 4**	6.6525	−10	22.6907
**FBG 5**	5.8837	−10	23.3302
**FBG 6**	3.2613	−10	25.5120
**FBG 7**	1.2898	−10	27.1522
**FBG 8**	0.5159	−10	27.7961
**FBG 9**	−0.3176	−10	28.4895
**FBG 10**	−1.3357	−10	29.3366

## Data Availability

The data presented in this study are available on request from the corresponding author.
